# Low birth weight risk prediction model: a prognostic study in the Birhan field site in Ethiopia

**DOI:** 10.7189/jogh.15.04209

**Published:** 2025-07-01

**Authors:** Achenef Asmamaw Muche, Yifru Berhan, Likelesh Lemma Baruda, Clara Pons-Duran, Bezawit Mesfin Hunegnaw, Robera Olana Fite, Kassahun Alemu Gelaye, Lisanu Taddesse, Delayehu Bekele, Getachew Tolera, Grace J Chan

**Affiliations:** 1 Health System and Reproductive Health Research Directorate, Ethiopian Public Health Institute, Addis Ababa, Ethiopia; 2 Department of Epidemiology and Biostatistics, Institute of Public Health, College of Medicine and Health Sciences, University of Gondar, Gondar, Ethiopia; 3 Department of Obstetrics and Gynecology, Saint Paul's Hospital Millennium Medical College, Addis Ababa, Ethiopia; 4 Maternal and Child Health Directorate, Federal Ministry of Health, Addis Ababa, Ethiopia; 5 Department of Epidemiology, Harvard T.H. Chan School of Public Health, Boston, Massachusetts, USA; 6 Department of Pediatrics and Child Health, St Paul’s Hospital Millennium Medical College, Addis Ababa, Ethiopia; 7 HaSET Maternal and Child Health Research Program, Addis Ababa, Ethiopia; 8 Deputy Director General Office for Research and Technology Transfer Directorate, Ethiopian Public Health Institute, Addis Ababa, Ethiopia; 9 Department of Pediatrics, Harvard Medical School, Boston, Massachusetts, USA

## Abstract

**Background:**

Pregnancy-related complications remain a global challenge, with low- and middle-income countries bearing the highest burden. Predicting the absolute risk of adverse birth outcomes will facilitate the delivery of early preventative and therapeutic interventions. We aimed to developed and internally validate a risk prediction model for low birth weight (LBW) in Ethiopia.

**Methods:**

We conducted a prognostic study using a prospective maternal and child health cohort in the Birhan field site, Amhara region, Ethiopia. We included all pregnant women with a live birth who had enrolled in the Birhan maternal and child health cohort between 2018 and 2021. We analysed data from 2076 live births. We first applied a multivariable logistic regression model to select variables for the risk prediction model, and used a classification and regression tree to select the most potent predictors. We presented the model with a nomogram suited to clinical use. We also calculated measures of risk prediction model accuracy, discrimination, and calibration, and used bootstrapping for internal validation. We assessed the clinical utility of the model using the decision curve analysis.

**Results:**

The incidence of LBW was 9.44% (95% confidence interval (CI) = 8.2, 10.8). We identified seven predictors: previous maternal complication, previous foetal complication, pregnancy induced hypertension, average maternal body weight, average diastolic blood pressure, preterm delivery, and gravidity. The prediction model had an area under the curve (AUC) of 0.67 (95% CI = 0.63, 0.72). After internal validation, the corrected discrimination AUC value was 0.64 (95% CI = 0.59, 0.68). The classification and regression tree identified four predictors: preterm, gravidity, average maternal body weight, and previous foetal complication, with a discriminative ability of 0.65 (95% CI = 0.61, 0.69). The decision curve analysis showed that the prediction model had high net benefit at different threshold probabilities in both the nomogram and the classification and regression tree.

**Conclusions:**

We developed a modestly accurate risk prediction model to identify pregnancies leading to LBW babies that could aid in early decision-making for prevention. This model is a crucial first step towards developing a clinical decision support tool to prompt early referral of women who are at high risk of having a LBW infant.

Hypertensive disorders of pregnancy, antepartum haemorrhage, postpartum haemorrhage, and low birth weight (LBW) are some of the most common pregnancy-related complications [1–3]. Among these, LBW remains a public health concern globally; over 20 million LBW babies are born each year, with 48% born in sub-Saharan Africa [4].

In 2018, a systematic review found the pooled estimate of LBW in Ethiopia to be 17.3% [5], while individual studies showed that the LBW burden varies significantly across the country, ranging from 8% to 54% [6,7]. Predictors of LBW include sociodemographic characteristics such as advanced maternal age [8,9], poor wealth index (income) [10], and lower level of maternal education [9–11]; obstetrical characteristics such as having twins [10], history of preterm delivery [12], having a caesarean section delivery [12], being primipara [9]; and pregnancy related complications such as pregnancy induced hypertension [9]. Various interventions have been implemented worldwide to prevent and reduce the prevalence of LBW, including nutritional interventions focusing on micronutrient supplementation and nutritional education [13], health system strengthening [14], balanced energy protein supplementation [15], consumption of low trans fatty acid diets [16], and dietary counseling [17].

The main purpose of the prognostic research or risk prediction models is to predict future outcomes for individuals, rather than groups [18]. However, the predictive accuracy of many risk stratification tools and prognostic models has been limited in Ethiopia, including those intended for the risk prediction for LBW that are aimed at identifying the absolute risk of individuals.

Developing a risk prediction model is an important first step in the development of a clinical decision support tool to prompt early referral of women at high-risk of delivering a LBW infant in low- and middle-income countries. Such a clinical tool could facilitate standard referral of these women before delivery, directing the limited resource of advanced neonatal care to infants at highest risk. In low-resource settings where prenatal ultrasound is infrequently available to evaluate foetal weight, identification of LBW in advance of delivery using predictive modelling could have a substantial impact on care.

Risk prediction models developed for clinical settings could help reduce preventable maternal and neonatal morbidity and mortality [19]. Individualised risk stratification could be used during the woman's first antenatal visit and adjusted for risk at all subsequent visits. The accuracy of a given risk prediction model is also crucial when informing clinical practice and policy aimed at improving neonatal health outcomes, including LBW. There have been few prior predictive models on LBW, so identifying high-risk pregnant women and their progeny could allow for early detection of LBW. Further, accuracy of existing models has been inconsistent, making them unseeable in guiding the most effective personalised management method [20–22]. We aimed to develop a validated risk prediction model for LBW in Birhan field site in Ethiopia.

## METHODS

### Study design, site, and population

We designed a prospective cohort study using a prognostic approach based on a sample of individuals from the Birhan Health and Demographic Surveillance System (BHDSS) open prospective pregnancy and birth cohort from December 2018 to January 2021 [23]. The BHDSS cohort was established in North Shewa Zone, Amhara Region, Ethiopia, to investigate the magnitude and causes of morbidity and mortality among pregnant and postpartum women and children. The site included 16 *kebeles* (the lowest administration unit) in two districts with approximately 18 933 households [24]. The Maternal and Child Health cohort is nested within the BHDSS and follows women through their pregnancy and postpartum period and their children from birth through the neonatal period up until two years of age. All pregnant women enrolled in the BHDSS cohort with complete pregnancy follow-up and a live birth were eligible for our study. Details of the Maternal and Child Health cohort follow-up process are available elsewhere [25].

### Variables

Our outcome variable was LBW, defined as a newborn with a birth weight of <2.5 kg [26]. We considered the following potential predictors of LBW: mother’s sociodemographic factors (age, wealth index, educational status, occupational status, marital status), behavioural factors (physical activity, alcohol intake), psychosocial factors (antenatal depression), obstetric characteristics (parity, history of miscarriage, stillbirth, and caesarean section), and nutritional status anaemia, dietary diversity), and laboratory tests (*e.g.* haemoglobin level). We used a multivariable prognostic risk prediction model to predict LBW after first determining potential predictors using bivariable logistic regression and selecting those with *P*-value ≤0.25. In the multivariable model itself, we considered predictors with a *P*-value <0.05 as LBW prognostic determinants.

### Data management

We determined the handling of missing data by the starting point of each predictor, where we excluded predictors missing at a rate of >50% from the prediction model [27]. Moreover, the prevalence of LBW in our sample was reduced even when we removed a relatively small number of individuals from the cohort. This suggests that individuals who experienced a LBW were more likely to have missing information, violating the assumption that data was missing at random. For this reason, we opted for multiple imputations to address this issue, as this is a valid technique that results in less bias than excluding all women with missing data [27]. To identify missing predictors that should be included in the imputation, we assumed that data was missing randomly and generated imputed data sets through performing multivariate imputation by chaining equations using the ‘mice’ package in *R* [28]. Variables with missing values ≤50% were imputed five times; we pooled these individual datasets and considered them in developing the risk prediction model.

### Data processing and analysis

We presented descriptive results as means, medians, standard deviations (SDs), interquartile quartile ranges (IQRs), and rates. We calculated and estimated the incidence of LBW for the study areas. To avoid losing prognostic information and to handle prognostic predictors, we used continuous variables rather than dichotomous variables. We evaluated the functional form of the association between continuous predictors and the outcome; when they were nonlinear, we presented them using fractional polynomials. Because the model contained several continuous variables, we used a multivariable fractional polynomial algorithm in each case [29].

### Risk prediction model development

We selected the prognostic determinants factors based on multivariable logistic regression analysis. The theoretical design was the incidence of low birth weight at a follow up time ‘t’ is a function of prognostic determinants ascertained at one time points before the occurrence of the LBW (‘t0’). The occurrence relation was determined per the formula: incidence of LBW = f (previous maternal complications + previous foetal complications + pregnancy-induced hypertension (PIH) + average maternal weight + average diastolic blood pressure + being preterm + gravidity).

We evaluated the discrimination probability of the risk prediction model according to the area under receiver operating characteristics curve (AUC) using the ‘pROC’ package in *R* [30]. The AUROC is the notation of decision plotting of true positive fraction (sensitivity) *vs*. false positive fraction (1 − specificity), with different cut points alongside the curve. Therefore, for any prediction technique to be meaningful, the AUC must be greater than 0.5 [31,32]. We assessed the model calibration or the plot of agreement between observed *vs*. predicted probability of an event using the ‘givitiR’ package in *R*, with an observed vs predicted probability overlie at 45°. We internally validated the model using the bootstrapping method with 1000 iterations (through sampling with replacement) to avoid overfitting of the model [33]. Bootstrapping method of internal validation is an ideal method for smaller sample sizes or for larger numbers of candidate predictors [34]. We assessed the model’s performance after bootstrapping, *i.e.* whether it was overfitted, by comparing its performance with that of the original model. We found no difference (zero optimism coefficient) between the apparent performance (actual model performance on samples) and true performance (model performance after internal validation).

### Clinical utility of the model

We performed a decision curve analysis (DCA) to complement the discrimination and calibration analysis to evaluate the prediction models that are crucial for clinical decision making [35]. The concept of the DCA involves the standard net benefit along the threshold probability, which is determined by the difference between benefits and costs. Here, the benefit refers to treating true positives (after correctly predicting a pregnant woman will have an LBW baby), while the cost is treating false positives (after incorrectly predicting a pregnant woman will have an LBW baby).

We evaluated the clinical utility of the model using DCA by determining the balance between the harm of a false-positive classification and the benefit of a true-positive classification [36]. We determined the net benefit of the developed model by a DCA plot using the ‘rmda’ package in *R*. We contrasted the model with the ‘treating all patients with suspected LBW’ and ‘not treating them at all’ scenarios.

### Nomogram development and cut-off point determination

We developed a nomogram to provide a simple, clinically applicable individual prediction of LBW [37] using a final, reduced model. Based on the developed clinical prediction model, we used the Youden index to estimate the cutoff point for predicted LBW probability and risk stratification. We then calculated the overall sensitivity, specificity, and positive and negative predictive value of our risk prediction model.

### Classification and regression tree

We used a classification and regression tree (CART) software to develop models that can classify subjects into various risk categories [38,39]. From seven variables used for risk prediction model development selected by multivariable analysis and nomogram development, the CART selected the four most potent predictors: being born preterm was the most important node that predicted low birth weight, followed by gravidity, average maternal body weight, and previous foetal complication.

We used *R*, version 4.1.3. (R Core Team, Vienna, Austria) for all of our analyses.

## RESULTS

### Sociodemographic characteristics

The BHDSS enrolled 2872 pregnant women in, of whom 2523 had live births. We excluded 447 pregnant women without data on their infant’s birth weight, leaving 2076 women with live births for analysis. Most of the participants were ethnic Amhara (91.4%). More than 92% of the participants were married. According to their wealth index, more than 40% of the participants were classified as poor. The mean maternal age was 27.2 years, with more than 60% of the participants being between 19 and 24 years of age (**Table 1**).

**Table 1:** Sociodemographic characteristics of study participants at Birhan Health and Demographic Surveillance System, Ethiopia, 2018–21

	Before imputation			After imputation		
	**LBW**			**LBW**		
	**Yes (%)**	**No (%)**	**Total (%)**	**Yes (%)**	**No (%)**	**Total (%)**
**Maternal age**						
≤18	13 (6.6)	99 (5.27)	112 (5.4)	13 (6.6)	99 (5.27)	112 (5.4)
19–24	115 (58.7)	1144 (60.9)	1259 (60.6)	115 (58.7)	1144 (60.9)	1259 (60.6)
25–34	46 (23.5)	356 (18.9)	402 (19.4)	46 (23.5)	356 (18.9)	402 (19.4)
≥35	22 (11.2)	281 (14.9)	303 (14.6)	22 (11.2)	281 (14.9)	303 (14.6)
**Marital status**						
Married	183 (93.4)	1741 (92.9)	1924 (92.9)	183 (93.4)	1747 (92.9)	1930 (93.0)
Not married	13 (6.6)	133 (7.1)	146 (7.1)	13 (6.6)	133 (7.1)	146 (7.0)
**Wealth index**						
Poorest	27 (13.8)	386 (20.5)	413 (19.9)	27 (13.8)	386 (20.5)	413 (19.9)
Poor	42 (21.4)	402 (21.4)	444 (21.4)	42 (21.4)	402 (21.4)	444 (21.4)
Medium	38 (19.4)	382 (20.3)	420 (20.2)	38 (19.4)	382 (20.3)	420 (20.2)
Rich	40 (20.4)	354 (18.8)	394 (18.9)	40 (20.4)	354 (18.8)	394 (18.9)
Richest	49 (25.0)	356 (18.9)	405 (19.5)	49 (25.0)	356 (18.9)	405 (19.5)
**Ethnicity**						
Amhara	180 (91.8)	1713 (91.3)	1893 (91.4)	180 (91.8)	1717 (91.3)	1897 (91.4)
Oromo	9 (4.6)	106 (5.7)	115 (5.6)	9 (4.6)	106 (5.6)	115 (5.5)
Others	7 (3.6)	57 (3.0)	64 (3.1)	7 (3.6)	57 (3.0)	64 (3.1)
**Education level**						
Primary	78 (73.6)	761 (79)	839 (78.5)	149 (76.0)	1505 (80.1)	1654 (79.7)
Secondary	23 (21.7)	145 (15.1)	168 (15.7)	34 (17.4)	249 (13.2)	283 (13.6)
Diploma and above	5 (4.7)	57 (5.9)	62 (5.8)	13 (6.6)	126 (6.7)	139 (6.7)
**Age at first pregnancy**						
15–19 years	70 (55.1)	799 (57.9)	869 (57.7)	113 (57.7)	1112 (59.2)	1225 (59.0)
≥20 years	57 (44.9)	579 (42.1)	636 (42.3)	83 (42.4)	768 (40.9)	851 (41.0)

LBW – low birth weight

### Clinical characteristics and obstetric comorbidities

The average weight of the participants taken during each visit ranged from 36 kg to 97.3 kg, with a mean of 57.4 kg (SD = 8.02). The average systolic blood pressure and diastolic blood pressure (DBP) were 103.1 mm Hg (SD = 9.6) and 65.7 mm Hg (SD = 6.9), respectively. More than 60% (1307) of the mothers were taking iron supplements at the time of enrollment. Most of the participants (n = 1507, 80.1%) had normal or above normal mid-upper arm circumferences (MUAC) measurement. Most of the participants (n = 1121, 74.5%) were multigravida.

Fasting during religious fasting seasons was practiced by 1777 (85.6%) participants. Most of the participants (>90%) had adequate dietary diversity. Antenatal depression was diagnosed among 279 (13.5%) of the participants, while 149 (9.9%) had psychosocial problems. Nausea and vomiting were experienced by 152 (7.33%) of the participants.

Complications and/or medical history from previous pregnancies were identified in 595 (28.7%) of participants, including preeclampsia/eclampsia, anaemia, diabetes, sexually transmitted disease, caesarean section, and miscarriage/abortion. Foetal complications from previous pregnancies were identified in 650 (31.3%) of participants before imputation, and in 750 (36.1%) after imputation, including neonatal death, neonatal jaundice, birth defects, early premature birth, LBW, and macrosomia (**Table 2**).

**Table 2:** Clinical factors and comorbidities, previous maternal pregnancy complications and previous pregnancy foetal/neonatal complications of study participants at Birhan Health and Demographic Surveillance System, Ethiopia, 2018–21

	Before imputation						After imputation			
	LBW*						**LBW**			
**Clinical factors and comorbidities**	**Yes (%)**	**No (%)**		**Total (%)**			**Yes (%)**		**No (%)**	**Total (%)**
Iron intake – yes	125 (63.8)	1182 (62.8)		1307 (62.9)			125 (63.8)		1182 (62.8)	1307 (62.9)
MUAC										
*Low*	40 (20.4)	373 (19.8)		413 (19.9)			40 (20.4)		373 (19.8)	413 (19.9)
*Normal and above*	156 (79.6)	1507 (80.1)		1663 (80.1)			156 (79.6)		1507 (80.1)	1663 (80.1)
Gravidity										
*Primigravida*	40 (31.5)	344 (24.9)		384 (25.5)			67 (34.2)		478 (25.4)	545 (26.6)
*Multigravida*	62 (48.8)	779 (56.5)		841 (55.9)			83 (42.4)		981 (52.2)	1064 (51.3)
*Grand multigravida*	25 (19.7)	225 (18.5)		280 (18.6)			46 (23.5)		421 (22.4)	467 (22.5)
Fasting during pregnancy – yes	157 (80.1)	1620 (86.2)		1777 (85.6)			157 (80.1)		1620 (86.2)	1777 (85.6)
Dietary diversity										
*Inadequate*	18 (9.4)	169 (9.1)		187 (9.2)			18 (9.2)		172 (9.2)	190 (9.2)
*Adequate*	173 (90.6)	1681 (90.1)		1854 (90.8)			178 (90.8)		1708 (90.8)	1886 (90.8)
Psychosocial problems – yes	10 (8.13)	139 (10.1)		149 (9.9)			21 (10.7)		201 (10.7)	222 (10.7)
Anaemia – yes	7 (7.7)	52 (4.9)		59 (5.2)			37 (18.9)		231 (12.3)	268 (12.9)
Nausea and vomiting – yes	11 (5.6)	141 (7.5)		152 (7.3)			11 (5.6)		141 (7.5)	152 (7.3)
Preterm – yes	63 (32.1)	244 (12.9)		307 (14.8)			63 (32.1)		245 (13.0)	307 (14.8)
Multiple birth – yes	7 (5.5)		45 (3.3)		52 (3.5)	12 (6.1)		62 (3.3)		74 (3.6)
APH – yes	0 (0)	3 (0.27)		3 (0.25)			13 (6.6)		79 (4.2)	92 (4.4)
PIH – yes	1 (1.02)	12 (1.1)		13 (1.1)			19 (9.7)		91 (4.8)	110 (5.3)
Preeclampsia/Eclampsia – yes	6 (3.1)	22 (1.2)		28 (1.4)			6 (3.1)		22 (1.2)	28 (1.4)
Anaemia – yes	40 (20.4)	283 (15.1)		323 (15.6)			40 (20.4)		283 (15.1)	323 (15.6)
Diabetes – yes	3 (1.53)	7 (0.37)		10 (0.48)			3 (1.53)		7 (0.37)	10 (0.48)
Caesarean section – yes	4 (3.2)	23 (1.7)		27 (1.8)			11 (5.6)		66 (3.5)	77 (3.7)
Miscarriage – yes	25 (20.33)	133 (9.7)		158 (10.6)			59 (30.1)		278 (14.8)	337 (16.2)
Abortion – yes	1 (0.8)	12 (0.9)		13 (0.9)			14 (7.1)		46 (2.5)	60 (2.9)
Early neonatal death – yes	10 (7.9)	74 (5.4)		84 (5.6)			18 (9.2)		132 (7.0)	150 (7.2)
Early premature birth	5 (3.9)	37 (2.7)		42 (2.8)			21 (10.7)		129 (6.9)	150 (7.2)
Neonatal jaundice – yes	3 (2.4)	4 (0.3)		7 (0.5)			23 (11.7)		79 (4.2)	102 (4.9)
Birth Defect – yes	0 (0)	4 (0.29)		4 (0.3)			20 (10.2)		127 (6.8)	147 (7.1)
LBW – yes	9 (9.5)	22 (2.1)		31 (2.6)			72 (36.7)		481 (25.6)	553 (26.6)
High birth weight – yes	4 (4.2)	25 (2.3)		29 (2.5)			32 (16.3)		293 (15.6)	325 (15.7)

APH – antepartum haemorrhage, LBW – low birth weight, MUAC – mid-upper arm circumference, PIH – pregnancy-induced hypertension, PPH – postpartum haemorrhage

### Incidence of LBW

We calculated the incidence as LBW as 9.44% (n/N = 196/2076) with a 95% CI of 8.2%, 10.8%.

### Model development for risk of LBW

We identified fourteen independent variables through our bivariable logistic regression as candidates for the multivariable logistic regression: first pregnancy, multiple birth, previous maternal complication, previous foetal complications, PIH, antepartum haemorrhage, average maternal body weight, anaemia, fasting during pregnancy, DBP, preterm, antenatal care visit, education level, and gravidity (multigravida and grand multipara). We classified those with with a *P*-value <0.05 as having a significant association with LBW. Predictors that showed significant associations with LBW were previous maternal complication, previous foetal complication, PIH, average weight, average DBP, preterm birth, and gravidity. The Hosmer-Lemeshow test had a *P* value of 0.37, implying a best fitted model (**Table 3**).

**Table 3:** Coefficients of predictor considered in the model to predict low birthweight at Birhan Health and Demographic Surveillance System, Ethiopia (n = 2076), 2018–21

	Bivariable logistic regression		Multivariable logistic regression*	
	**Coefficient (95% CI)**	***P*-value**	**Coefficient (95% CI)**	***P*-value**
**First pregnancy**				
No	ref		ref	
Yes	0.399 (−0.09, 0.71)	0.012	0.375 (−0.01, 0.76)	0.056
**Previous maternal complication**				
No	ref		ref	
Yes	0.31 (0.007, 0.61)	0.052	0.389 (0.036, 0.734)	0.029
**Average DBP**	0.029 (−0.009, 0.049)	0.005	0.027 (0.005, 0.04)	0.015
**Previous foetal complication**				
No	ref		ref	
Yes	0.496 (0.199, 0.79)	0.001	0.415 (0.095, 0.732)	0.01
**Average maternal weight**	−0.024 (−0.043, −0.005)	0.015	−0.02 (−0.046, −0.004)	0.016
**Multiple birth**				
No	ref		ref	
Yes	0.65 (−0.03, 1.24)	0.046	0.357 (−0.38, 1.03)	0.319
**PIH**				
No	ref		ref	
Yes	0.75 (0.20, 1.24)	0.0047	0.694 (0.04, 1.30)	0.03
**APH**				
No	ref		ref	
Yes	0.48 (−0.16, 1.05)	0.119	−0.54 (−1.38, 0.252)	0.19
**Anaemia**				
No	ref		ref	
Yes	0.51 (0.11, 0.88)	0.009	0.39 (−0.09, 0.85)	0.104
**Fasting in pregnancy**				
No	ref		ref	
Yes	−0.43 (−0.80, −0.05)	0.022	−0.25 (−0.64, 0.16)	0.22
**Preterm**				
No	ref		ref	
Yes	1.15 (0.81, 1.48)	<0.001	1.06 (0.70, 1.42)	<0.001
**Antenatal care visit**				
No	ref		ref	
Yes	−0.34 (−0.75, 0.10)	0.12	−0.22 (−0.67, 0.26)	0.35
**Education level**				
Primary	ref		ref	
Secondary	0.32 (−0.08, 0.71)	0.11	0.37 (−0.06, 0.79)	0.087
Diploma and above	0.04 (−0.59, 0.60)	0.89	0.24 (−0.42, 0.83)	0.45
**Gravidity**				
Primigravida	ref		ref	
Multigravida	−0.50 (−0.84, −0.16)	0.036	−0.577 (−0.94, −0.21)	0.009
Grand multigravida	−0.25 (−0.65, 0.14)	0.22	−0.52 (−0.99, −0.06)	0.029
**Intercept**			−2.89 (−4.77, −1.02)	0.0024

APH – antepartum haemorrhage, DBP – diastolic blood pressure, PIH – pregnancy-induced hypertension, ref – reference

*p(LBW) = −2.89 + 0.39 (previous maternal complication) + 0.027 (average DBP) + 0.415 (previous foetal complication) − 0.02 (average maternal weight) + 0.694 (PIH) + 1.06 (preterm) − 0.577 (multigravida) − 0.52 (grand multigravida).

### CART analysis

**Figure 1:**
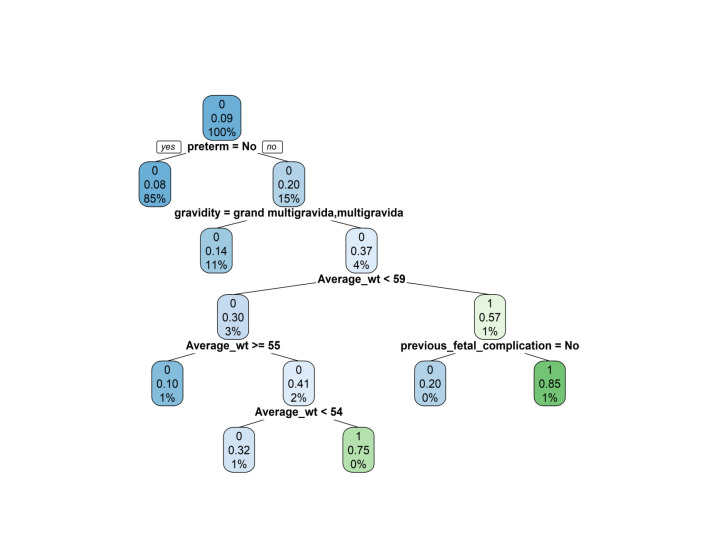
Classification and regression tree to predict low birth weight at Birhan Health and Demographic Surveillance System, Ethiopia, 2018**–**21. wt – weight.

From seven variables used for risk prediction model development selected by multivariable analysis, the CART selected the four most potent predictors: being born preterm was the most important node that predicted low birth weight, followed by gravidity, average maternal body weight, and previous foetal complication. The root node began with preterm and contained the total population divided into two groups. There were five decision nodes, where each sub node splits into two sub nodes. There were seven leaf nodes, or terminal nodes. The first number for each node represented the status of the outcome LBW (0 = no and 1 = yes). The second number showed misclassification of the participants for being in that specific node. The third number showed the total proportion of population in each node (**Figure 1**).

Of the preterm pregnant women, 8% were misclassified as not at risk from LBW and 85% were at risk for LBW babies in resides in this node in same node (**Figure 1**). The seventh terminal node showed that, among those who are preterm, primigravida, had an average maternal weight ≤59 kg, and had previous foetal complication history, 85% were correctly classified as at risk of LBW.

### Model performance

**Figure 2:**
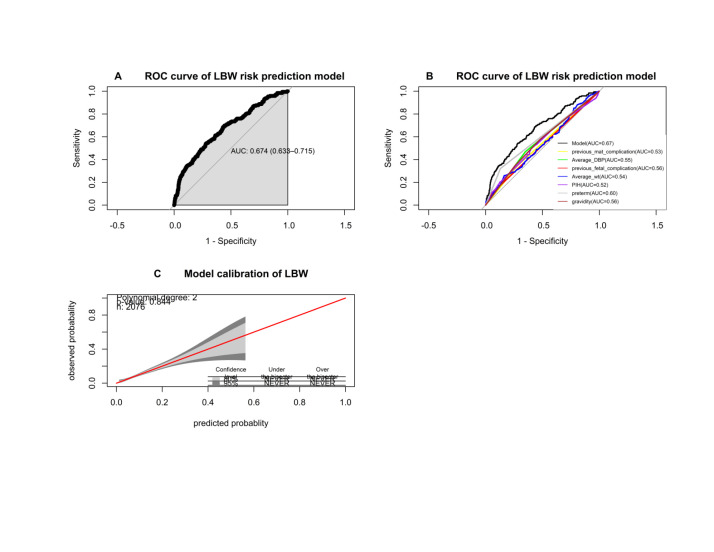
Discrimination ability of the model (2A), contribution of each predictor to the model (2B) and calibration plot to the model (2C) to predict low birth weight at the Birhan Health and Demographic Surveillance System, Ethiopia, 2018–21.

The AUC had a good discrimination ability to differentiate if newborns would be LBW or not. The nomogram-based multivariable LBW risk prediction model had an AUC of 0.674, (95% CI = 0.633, 0.715) (**Figure 2**, Panel A). The predictors that indicated LBW included previous maternal complication (AUC = 0.53; 95% CI = 0.50, 0.57), average DBP (AUC = 0.55; 95% CI = 0.51, 0.59), previous foetal complication (AUC = 0.56; 95% CI = 0.52, 0.60), average weight (AUC = 0.54; 95% CI = 0.49, 0.58), PIH (AUC = 0.52; 95% CI = 0.50, 0.55), preterm birth (AUC = 0.60; 95% CI = 0.56, 0.63), and gravidity (AUC = 0.56; 95% CI = 0.52, 0.59) (**Figure 2**, Panel B). The performance of the CART for predicting LBW was an AUC of 0.648 (95% CI = 0.606, 0.6902), while the Hosmer-Lemeshow goodness of fit test showed it to be well-calibrated (*P* = 0.389) (Figure S1 in the **Online Supplementary Document****).** The calibration belt did not show evidence of a lack of calibration. The bisector was contained by the calibration belt in the whole 0–1 range; it was neither below nor above the bisector. This implies that the predictions were not larger or smaller than the actual observed rates of LBW. The *P*-value of 0.844 in the model indicated that the calibration of the model on the development sample was acceptable (**Figure 2**, Panel C).

Good discrimination ability is indicated at a point where the overlap between the positive and negative cases is low. The overlapping increases when the probability of LBW is low. Therefore, the prediction density plot showed that the models had no perfect predictive ability of classifying cases and non-cases (Figure S2 in the **Online Supplementary Document****).**

### Cut-off point for probability of LBW

Based on the beta coefficients of the predictors used for the nomogram model development, the optimal cut-off point for the predicted probability of risk of LBW was found to be 0.099, determined by the Youden Index. The cut-off point for the maximum Youden Index (sensitivity + specificity − 1) was taken as the optimum cut-off point to classify as low and high risk of LBW. The sensitivity, specificity, positive predictive value, negative predictive value (NPV), and accuracy were 54.1%, 72.1%, 16.8%, 93.7%, and 63.1%, respectively.

### Internal validation

The model was validated internally by bootstrapping with 1000 repetitions with replacement. It had an optimism corrected model accuracy for discrimination at an AUC of 0.639 (95% CI = 0.597, 0.681), with sufficient prediction discrimination and a calibration analysis. The performance of the CART after bootstrapping was the same as the developed CART model, showing good accuracy with an AUC of 0.645 (95% CI = 0.603, 0.688) and comparable with less optimism coefficients found to be 0.0026, which indicates less likely overfitted model and well calibrated model (*P* = 0.389). The average calculated value of optimism to the performance model was 0.0013, which was the smaller value, as the discriminatory power of the model before and after internal validation had no significant difference (Figure S3 in the **Online Supplementary Document****).**

### DCA

The DCA assessed the clinical practicability of the risk prediction model. The DCA plot demonstrated the net benefit of using the developed nomogram model to estimate the future risk of an individual LBW outcome if the threshold probability is 0.05 or more, as opposed to a treat all/treat none strategy. The DCA further showed that the established model for early risk classification of pregnant women for LBW has a higher clinical benefit than treating none and treating all pregnant women across a wide range of threshold probability (Figure S4 in the **Online Supplementary Document****).**

### Nomogram

**Figure 3:**
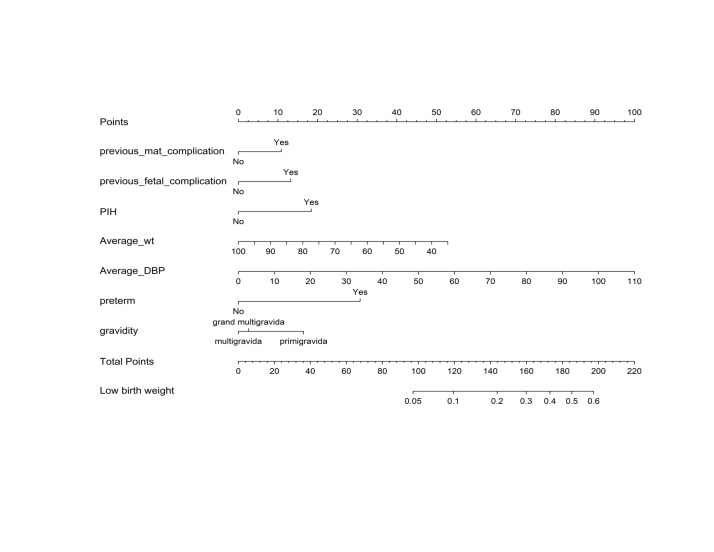
Nomogram to predict low birth weight among live births at the Birhan Health and Demographic Surveillance System, Ethiopia, 2018**–**21. The point score of each risk factor can be calculated separately by reading the score above the factor vertically. Then, the points from each variable value were summed. The sum on the total points scale was located and vertically projected onto the bottom axis, and then, a personalised LBW risk was obtained.

We used statistically significant predictors from the multivariable logistic regression to develop a nomogram to calculate the individual risk of LBW among live births. We assigned each category of the variables a score to measure future risks. We then gave the observed value of each predictor a certain number of points by drawing a vertical line towards the top of the nomogram. We indicated the sum of the points for each predictor at the bottom and labelled it as ‘total points’. The decimal points below the total points correspond to the individual’s risk of LBW. For example, we estimated the probability of LBW to be 10.2% in our sample, with a total score of 121 (**Figure 3**).

## DISCUSSION

In our analysis, predictors significantly associated with LBW were maternal and foetal complications from previous pregnancy, average DBP, average maternal weight, PIH, preterm birth, and gravidity. Furthermore, the nomogram we developed here showed clinical usefulness. We developed a modestly effective model that clinicians can use to identify 54% of pregnant women who are at risk for a LBW birth, making it easier to deliver preventative and therapeutic interventions to high-risk women.

The incidence of LBW in the BHDSS study site remains high, which is consistent with the evidence from other contexts [4,40,41]. We found that the incidence of LBW in our sample also aligned with that found in studies conducted in other locations in Ethiopia: Axum (9.9%) [42], Arsi (9.1%) [43] and Hosanna (9.8%) [44]. However, it was lower than the national pooled prevalence of 14.1% [45], 21% in Dire Dawa [46], 12.5% in Gondar [47], 16.5% in Sidama [48], and 27% in Mekelle [49]. These results could be explained by improved antenatl care services, relatively fewer pregnancies among women aged <18, and lower preterm births in our setting.

Predictors with a significant association with LBW which we used to develop the risk prediction model included maternal complications from previous pregnancy, average DBP, foetal complication from previous pregnancy, average maternal weight, PIH, preterm birth, and gravidity. This combination resulted in AUCs of 0.674 for development and 0.639 for validation, and provided evidence that the model is 64% capable of differentiating between pregnant women who will have and will not have a LBW newborn. The discriminative ability of our model is lower than of those developed in similar studies from Ethiopia with an AUC of 0.83 [39], as well as those in India, which had an AUC of 0.79 [40]. The discrepancy might be explained by the difference in predictors used for model development and the larger proportion of LBW babies. The Ethiopian study utilised age at pregnancy, underweight, anaemia, height, gravidity, presence of comorbidity, and had a LBW prevalence of 21.9%. The study conducted in India [40] used factors such as inadequate maternal diets (such as insufficient protein intake during pregnancy), having previous preterm labour, having a LBW baby, having anaemia in pregnancy, and having a history of substance abuse (*e.g.* smoking) as predictors in the development of the risk prediction model. It also reported a high prevalence of LBW (50%) [40]. These factors could possibly explain the discrepancy in the model accuracy level.

We developed a model for quantifying the individual risk of giving birth to a LBW baby by identifying easy to collect and compile predictors. Clinicians can determine the likelihood of LBW by adding up to seven prognostic predictors to the nomogram to see a graphical display of individual risk. The decision curve analysis proved the value of applying the models in clinical setting by showing that, if the threshold probability is >5%, the application of the model is better than a treat all/treat none strategy. In low-resource settings where prenatal ultrasound is infrequently available to evaluate foetal weight, identification of LBW in advance of delivery using risk prediction model could substantially impact early decision making and care. Developing an easy, non-invasive, useful, and precise model for determining the risk of LBW in different periods of pregnancy is crucial in general, but particularly in resource-limiting settings where there is a shortage of imaging equipment and trained personnel to diagnose or predict foetal growth restrictions earlier in the gestation period.

The strength of our study lie in its longitudinal nature and the fact that we built our risk prediction model with a sufficient number of events per predictor. We used multiple imputations to address missing data, which is a valid technique that results in less bias than excluding all women with missing data [27]. In addition, we constructed the LBW risk prediction model from easily available, measurable, and applicable predictors used to predict future risk of low birth weight, making it applicable in clinical settings. However, we did not perform external validation, which may affect the generalisability of our findings. Furthermore, we did not address the potential influence of social or cultural factors, such as healthcare access or health-seeking behaviours, which could impact LBW risk. We also had a lower AUC for our model which, while acceptable, requires further clinical parameters for risk prediction of LBW.

## CONCLUSIONS

We identified easy to collect predictors for the early identification of pregnant women who are at risk of delivering a LBW baby and developed a risk prediction model. The resulting model has clinical utility advantages and can prevent LBW-related complications by identifying those at risk for referral to centres with the necessary facilities. This is a crucial first step towards developing a clinical decision support tool to prompt early referral of women who are at high risk of having a LBW infant, or more specifically, a clinical decision support tool incorporating maternal characteristics and clinical parameters that would enhance care by standardising referral decisions related to the anticipated delivery of a LBW infant in Ethiopia. We recommend that obstetricians use the model to predict LBW and manage it appropriately. However, we did not externally validate the model was using an independent dataset, limiting its prediction capability when applied to other contexts. We recommend that future research addresses this gap before the model is used in other settings.

## Additional material


Online Supplementary Document
