# Supplementary material for: Low birth weight risk prediction model: a prognostic study in the Birhan field site in Ethiopia
Source: J Glob Health. 2025 Jul 1;15:04209. doi: 10.7189/jogh.15.04209 (PMC12210211; doi:10.7189/jogh.15.04209)
Supplement: Online Supplementary Document [file jogh-15-04209-s001.pdf]

Supplement to: Muche AA, Berhan Y, Baruda LL, Pons-Duran C, Hunegnaw BM, Fite RO, Gelaye KA, Tadesse L, Bekele D, Tolera G, Chan GJ. Low birth weight risk prediction model: a prognostic study in the Birhan field site in Ethiopia. J Glob Health. 2025:15:04209.

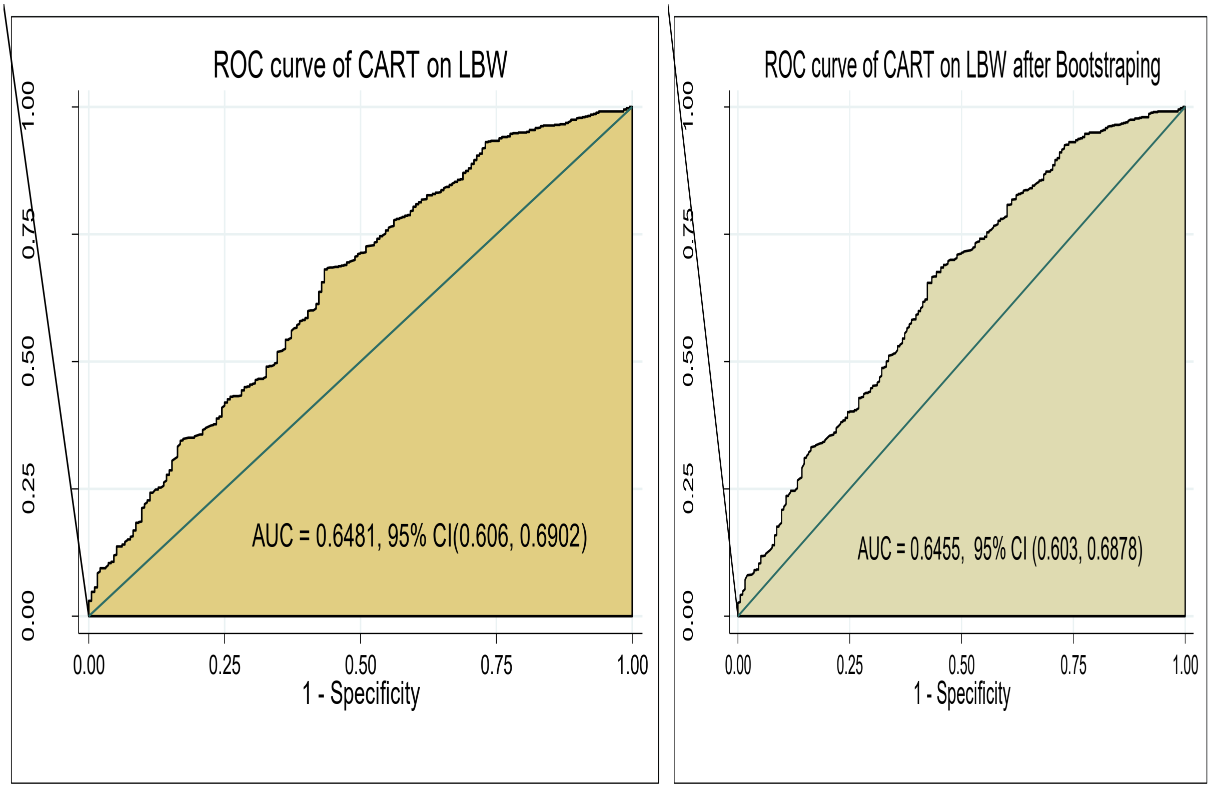

Figure S1. Discrimination ability of classification and regression tree (left) and after internal validation (right) to predict low birth weight at the Birhan Health and Demographic Surveillance System, Ethiopia: 2018-2021.

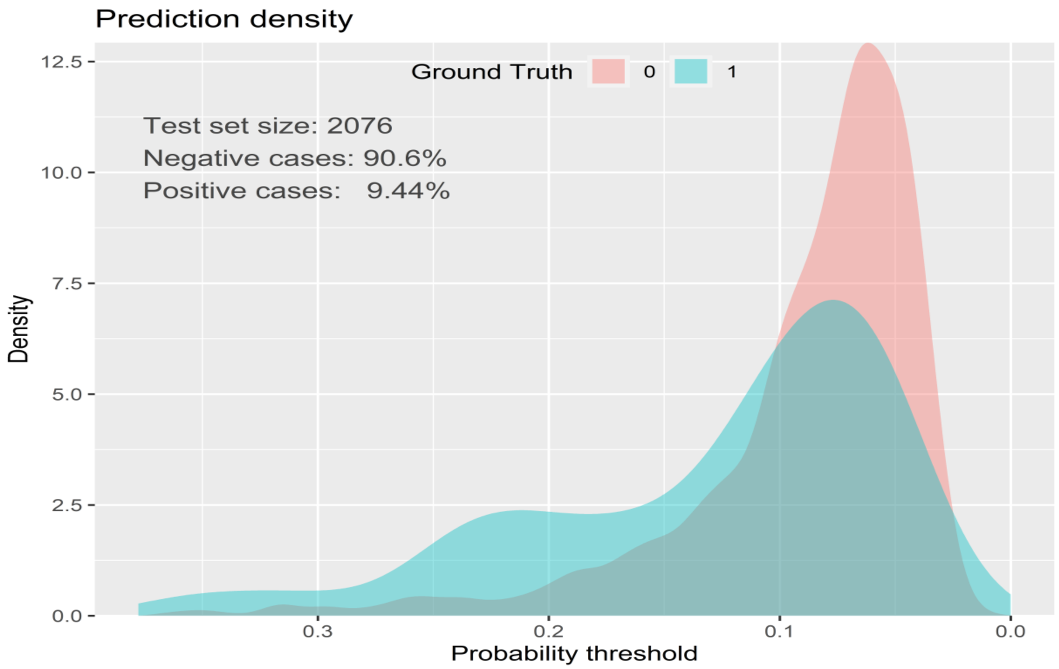

Figure S2. Prediction density for the prediction of low birth weight at the Birhan Health and Demographic Surveillance System, Ethiopia, 2018–21.

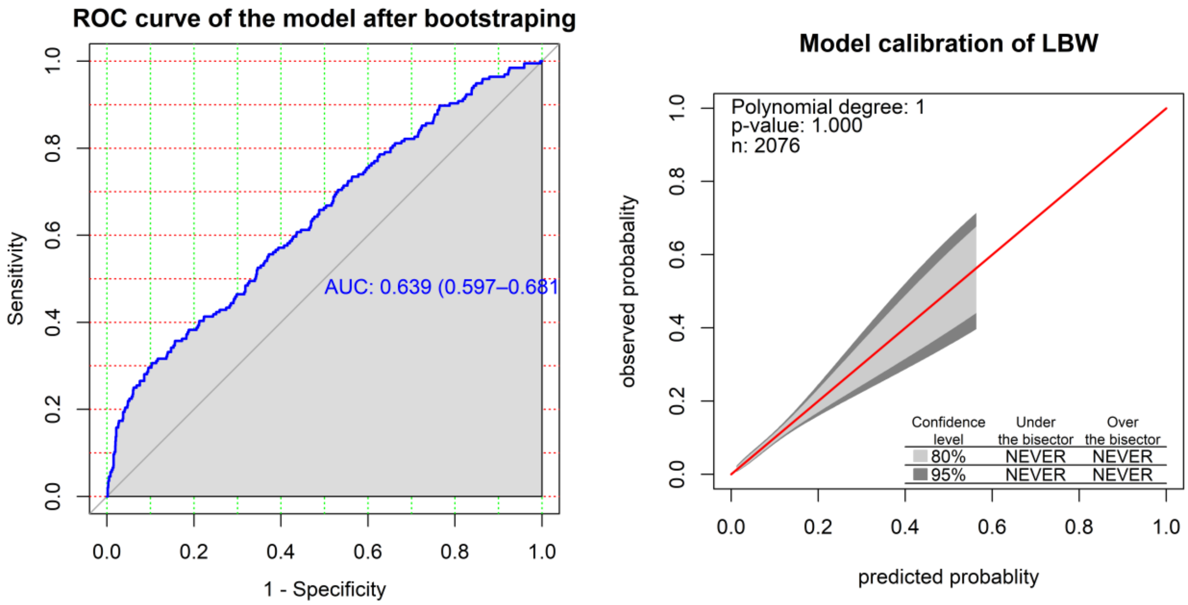

Figure S3. Optimism corrected discrimination (left) and calibration (right) of the nomogram based risk prediction model after internal validation to predict low birth weight at the Birhan Health and Demographic Surveillance System, Ethiopia, 2018–21.

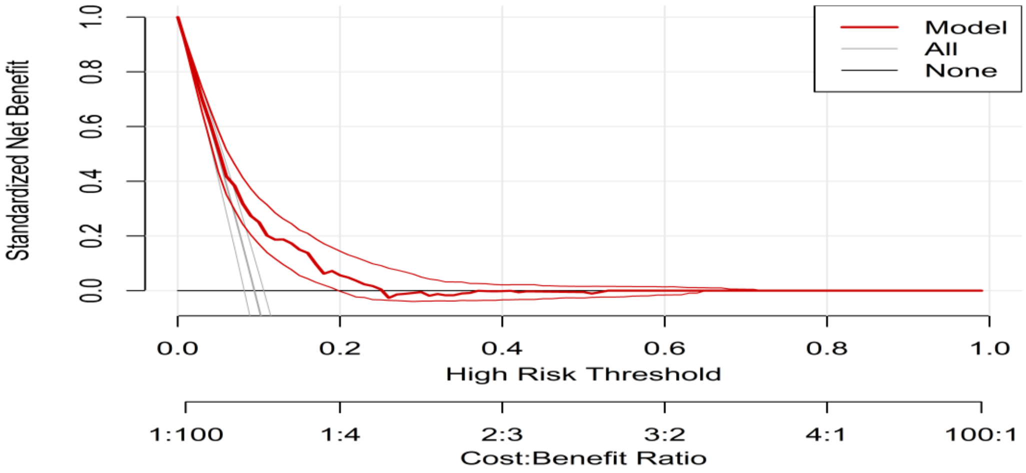

Figure S4. Decision curve analysis for the risk prediction model for low birth weight at the Birhan Health and Demographic Surveillance System, Ethiopia, 2018–21.
